# The Embodiment of Success and Failure as Forward versus Backward Movements

**DOI:** 10.1371/journal.pone.0117285

**Published:** 2015-02-06

**Authors:** Michael D. Robinson, Adam K. Fetterman

**Affiliations:** 1 North Dakota State University, Fargo, North Dakota, United States of America; 2 Knowledge Media Center, Tübingen, Germany; VU University Amsterdam, NETHERLANDS

## Abstract

People often speak of success (e.g., “advance”) and failure (e.g., “setback”) as if they were forward versus backward movements through space. Two experiments sought to examine whether grounded associations of this type influence motor behavior. In Experiment 1, participants categorized success versus failure words by moving a joystick forward or backward. Failure categorizations were faster when moving backward, whereas success categorizations were faster when moving forward. Experiment 2 removed the requirement to categorize stimuli and used a word rehearsal task instead. Even without Experiment 1’s response procedures, a similar cross-over interaction was obtained (e.g., failure memorizations sped backward movements relative to forward ones). The findings are novel yet consistent with theories of embodied cognition and self-regulation.

## Introduction

A great deal of our computational power derives from the ability to represent abstract concepts—concepts lacking a direct sensory component and somewhat divorced from particulars of time and place. For example, self psychologists have attributed the sophistication of modern cultural practices to the development of an abstract, symbolic sense of self, one that transcends environmental contingencies [1]. In classic cognitive theories, abstract concepts (e.g., of self or meaning) are represented in a way that can be considered disembodied. That is, their representational format and use is thought to be independent of the particular ways we perceive information and interact with the environment (e.g., [2–3]). This way of characterizing abstract thought can be contrasted with the embodied cognition perspective, whereby even abstract thoughts retain some connections to sensory and motoric forms of representation [4–5].

Support for the embodied cognition perspective has come from a number of studies, the majority of which have used linguistic materials because of their links to conceptual processing (for a review, see [6]). People are faster to verify that a pictured object had been described in a previous sentence when the orientation of the pictured object matches that implied by the sentence [7]. People are faster to verify that word objects possess certain sensory qualities (e.g., leaves-rustle) when the sensory modality matches rather than mismatches across consecutive trials [8]. Performing an action (e.g., grasping) facilitates the recognition of phrases describing that action [9]. And there is evidence that sensory areas of the brain are activated when people verify that linguistic objects possess certain sensory properties [10]. Perceptual and motor simulations, that is, often occur when people seek to understand linguistic materials whose corresponding referents have perceptual or motor features [11].

What about abstract concepts, though—those without inherent perceptual or motor features? Pecher, Boot, and Van Dantzig [12] make the case that we have less knowledge concerning the possible embodiment of these concepts, but there are relevant theories. Conceptual metaphor theory [13–14], in particular, contends that abstract concepts (e.g., the self or one’s life) are difficult to understand without recruiting a more concrete domain by way of comparison. These links of abstract target and concrete source are metaphoric and can be observed in the language that people often use to describe abstract concepts [15]. For example, feeling happy is likened to feeling *up* and feeling sad is likened to feeling *down* [16]. Although linguistic metaphors can be fairly elaborate [15], they are often organized by relatively basic sensory-motor experiences [17] such as seeing something that is lighter or darker, higher or lower, or moving forward or backward [18–19].

In the present research, we examine the possible embodiment of success and failure concepts. These concepts are fundamental to goal systems and they are fairly abstract [20]. Nonetheless, metaphoric language often suggests a spatial mapping onto forward versus backward movements [14]. Having successes can be described as *moving forward*—i.e., *toward* one’s goals. When things are not going well, by contrast, we are often *moving backward*, having *setbacks*, and feeling very *behind*. Much of this language probably derives from a journey metaphor whereby desired outcomes are destinations ahead of us and progress consists of moving toward them rather than away from them [21]. In support of this idea, Natanzon and Ferguson [22] found that cues to forward motion primed achievement motivation and Landau et al. [20] found that imagining oneself on a forward path through college boosted academic intentions.

The journey metaphor reflects more basic considerations, however. Our eyes are in the front of our heads and our bodies move more smoothly forward rather than backward [23]. As a result, we tend to move forward to obtain desired objects in the environment [23–24]. By contrast, backward movements may typically occur when there is an environmental object that we want to move away from while still seeing it [24]. These dynamics are fundamental enough that a number of self-regulation theories consider approach motivation in terms of a forward movement and avoidance motivation in terms of a backward movement [25–27]. From this self-regulation perspective, too, it would be somewhat natural to think of (goal-related) success as a forward motion and failure as a backward motion [21,28].

We sought to examine these ideas in cognitive-experimental terms. To focus on the abstract concepts of success and failure, word stimuli were used [29]. To examine the grounding of these concepts, success and failure words were treated as primes in the paradigms [30]. To determine whether these concepts are grounded in representations of movement, a variation of the joystick task [31] was used. Two experiments were performed, both for the sake of replication and to examine possible limiting conditions. In each case, we expected factorial interactions between concept activation (success versus failure) and movement direction (forward versus backward).

## Experiment 1

Experiment 1 consisted of a movement-related categorization task modeled after [32]. Participants were asked to categorize success or failure words by moving a joystick forward (one category of words) or backward (the other category of words), with movement directions counterbalanced across task blocks. If there is an embodied link between goal status (success versus failure) and movement direction (forward versus backward), there should be an interaction between word category and movement direction. For example, success words should be categorized faster with a forward than backward motion.

## Method

### Sample Size Considerations

The focus of the investigation was novel, but previous research with movement-related paradigms (e.g., [33]) led us to expect medium effect sizes. We aimed for samples of 80–120, which should be adequately powered for effects of this magnitude in within-subject designs [34]. The experiments were run for a fixed period of time, a period of time matched to the sample sizes desired and based on past research with the same participant pool at the same institution. The exact number of participants, though, depended on sign-up and show-up rates, which differed by experiment.

### Participants and General Procedures

Seventy-six (36 female) undergraduate students from North Dakota State University received research credit for their psychology classes. They arrived to a laboratory in groups of 6 or less and completed the experimental task on personal computers separated by dividers. Both experiments were approved by the Institutional Review Board of North Dakota State University. Finally, all participants read and signed a physical informed consent form. The data for these experiments are available upon request by emailing the authors.

### Experimental Task

Instructions and Blocks.

Participants were instructed to categorize words in terms of whether they signified success or failure. They were to make these categorizations by moving a joystick lever forward or backward. For 3 of 6 blocks, success (failure) words required a forward (backward) movement. For the other 3 of the 6 blocks, these mappings were reversed. Blocks were randomly ordered on a per-participant basis.

Success versus Failure Stimuli.

Stimuli were selected on the basis of prior norms (e.g., [35]) and Thesauri, largely centering on the words *succeed* and *fail*. It was deemed useful to focus on verbs because verbs are particularly indicative of self-regulation in action [36]. On the basis of such considerations, 10 success-related (accomplish, progress, succeed, win, thrive, complete, attain, achieve, advance, & triumph) and 10 failure-related (fail, lose, recede, flop, blunder, fall, flounder, decline, backslide, & lapse) words were chosen.

Equipment.

Screen resolution was set to 1,280 by 1,024 pixels. Movements were made with a Saitek Aviator-01 dual throttle joystick.

Trial Procedures.

There were 120 trials. Each began with a randomly selected word presented at center screen. It disappeared upon movement onset, a procedure designed to increase the priming-related (concept to movement) features of the task while removing screen clutter. Movement time was the length of time that elapsed from the presentation of the word until the joystick cursor had been moved to the top (F) or bottom (B) of the computer screen. Movements made in the wrong direction were penalized with a visual error message. After a movement had been completed, participants were instructed to return to center screen, after which there was a 300 ms blank delay until the next trial began.

Data Preparation.

Stimuli were perceived to belong to their intended categories in that movements were made in the correct direction 93.0% of the time. Trials on which movements were made in the wrong direction were deleted. Movement times were positively skewed and we therefore log-transformed them for analysis purposes. Then, to reduce the impact of outliers, log-transformed movement times 2.5 *SD*s above or below the overall log latency mean were replaced with such 2.5 outlier values [37]. Subsequently, data were averaged for cells of the 2 (word category) by 2 (movement direction) experimental design.

## Results

**Fig 1:**
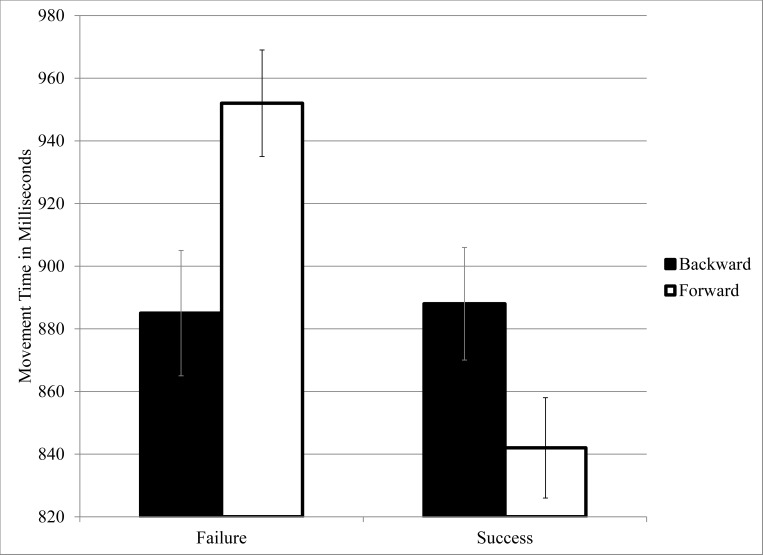
Movement Times with Standard Errors as a Function of Word Category (Success versus Failure) and Movement Direction (Forward versus Backward), Experiment 1.

Log-transformed movement times were analyzed as a function of word category (success versus failure) and movement direction (forward versus backward) in a repeated-measures ANOVA. There was a main effect for Word Category, *F* (1, 75) = 64.81, *p* < .01, partial eta squared (PES) = .46, such that movements tended to be faster on trials with success primes (*M* = 865 ms) than failure primes (*M* = 919 ms), despite the slightly longer word length of the former stimuli (*M* = 6.80 letters) relative to the latter (*M* = 5.80). There was also a main effect for Movement Direction, *F* (1, 75) = 6.00, *p* = .02, PES = .07, with backward movements faster (*M* = 887 ms) than forward movements (*M* = 897 ms). No such tendency was observed in Experiment 2, however. Of most pertinence, there was a Word Category by Movement Direction interaction, *F* (1, 75) = 20.23, *p* < .01, PES = .21 (a large effect size). Means for the interaction are displayed in Fig. 1 and they indicate a hypothesis-consistent cross-over pattern. Specifically, failure primes triggered faster backward than forward movements, *F* (1, 75) = 24.17, *p* < .01, PES = .24, whereas success primes triggered faster forward than backward movements, *F* (1, 75) = 8.27, *p* < .01, PES = .10.

We should also note that there was also a Word Category by Movement Direction interaction for accuracy rates, *F* (1, 75) = 20.89, *p* < .01, PES = .22. Accuracy rates were comparable for success (*M* = 94.20%) and failure (*M* = 94.77%) words when moving backward, but higher for success (*M* = 95.03%) than failure (*M* = 89.97%) words when moving forward. Accuracy rates tended to be high, however, and therefore what is emphasized is the lack of a speed-accuracy tradeoff for the movement time interaction reported above.

Additionally, a reviewer noted that 2 of the success words (advance & progress) and 2 of the failure words (backslide & recede) could refer to literal forward or backward motions. That this is true in a sense contributes to the paper’s embodied analysis, but one would also hope that the hypothesized interaction is not entirely reliant on polysemy of this type. It was not in that the Word Category by Movement Direction interaction remained significant when deleting trials involving these word stimuli, *F* (1, 75) = 19.61, *p* < .01, PES = .21.

## Discussion and Experiment 2

Experiment 1 provided novel evidence for the idea that abstract representations of success and failure are grounded on simulations of forward versus backward movement. Moreover, Experiment 1 used task procedures that have been recommended for their sensitivity and validity in the approach/avoidance literature [31]. Specifically, this literature recommends procedures in which people categorize stimuli by making movements, thereby introducing elements of a stimulus-response compatibility type [32]. Just as in this literature, though, one might want to determine what happens when such potential contributions are removed [38]. The posited grounding of success and failure as forward versus backward motion, and therefore the prime type by movement direction interaction, should still be evident under these circumstances [29]. To investigate this possibility, Experiment 2 cued movement direction in a manner independent of whether success or failure concepts were activated [39]. In addition, Experiment 2 removed a binary choice feature from the prime task, one that can contribute to structural stimulus-response compatibility links under certain circumstances [40]. We did this by using a word rehearsal task on the primes (see below).

## Method

### Participants and General Procedures

A new group of 128 (69 female) undergraduate students arrived to the laboratory in groups of 6 or less and completed the second experiment.

### Experimental Task

Instructions.

Participants were told that we were interested in abilities to alternate between two very different tasks [41]. The first involved rehearsing a word and the second involved moving a joystick forward or backward depending on whether the letter “F” (forward) or “B” (backward) was presented.

Stimuli and Equipment

The stimuli and equipment described in Experiment 1 were also used in Experiment 2. In addition, participants wore voicekey headphones.

Trial Procedures.

There was no manipulation of response mappings in Experiment 2 and therefore all trials occurred within a single block. Levels of the two experimental factors were randomly assigned at the trial level. Each trial began with a centered word, presented for 1500 ms, to be rehearsed during the trial. After the 1500 ms, the word disappeared and did not reappear. There was then a 200 ms blank delay until the letter “F” (forward) or “B” (backward) was presented at center screen. Movements initiated in the wrong direction were penalized by an error message and relevant data were deleted. For movements in the correct direction, the computer program recorded the time that elapsed between the movement letter and completion of the movement, defined in terms of moving the joystick cursor toward the very top (F) or bottom (B) of the computer screen. Participants then returned the joystick cursor to center screen, following which the word initially presented was spoken into a voicekey microphone. That is, there was no binary choice task [40] involving the primes. Subsequently, there was a 300 ms delay before the next trial began.

Data Preparation.

Movements in the correct direction were observed for 92.7% of the trials. Such movement times were positively skewed and therefore transformed in the same way as in Experiment 1 (e.g., log-transformation). Data were then averaged for cells of the 2 (success versus failure) by 2 (forward versus backward) design.

## Results

**Fig 2:**
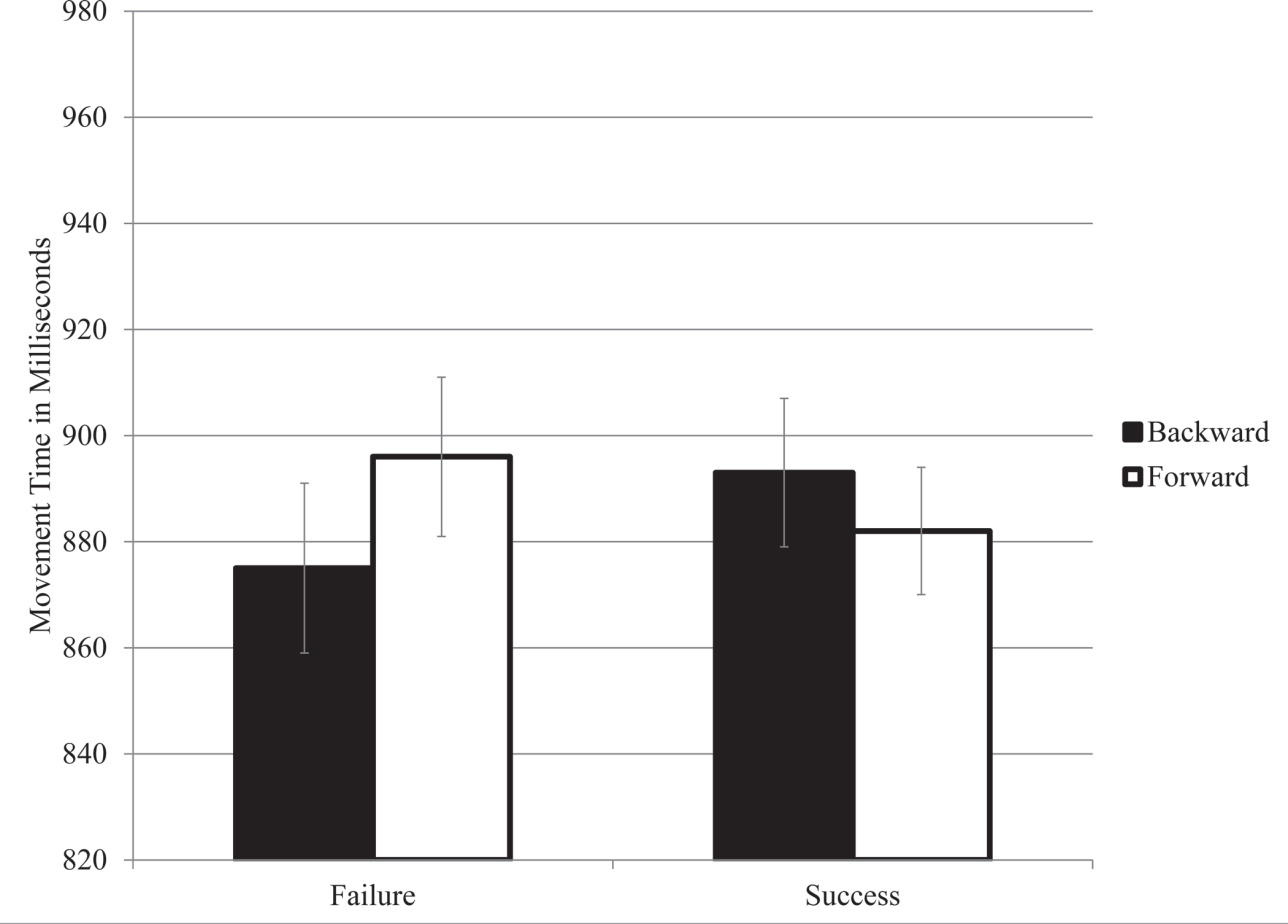
Movement Times with Standard Errors as a Function of Prime Category (Success versus Failure) and Movement Direction (Forward versus Backward), Experiment 2.

A cross-over interaction of the type found in the first experiment was hypothesized. It should be of lesser magnitude than in Experiment 1 given the removal of response compatibility elements, but it was still hypothesized to be significant. This hypothesis was tested in a 2 (Prime Category) by 2 (Movement Direction) repeated-measures ANOVA, with log-transformed movement times as the dependent measure. Main effects for Prime Category and Movement Direction were not significant, *F*s < 1. There was, however, a Prime Category by Movement Direction interaction, *F* (1, 127) = 14.25, *p* < .01, PES = .10 (in between a medium and large effect size). Millisecond means for this interaction are reported in Fig. 2 and they indicate that that the cross-over pattern of the first experiment was replicated. Although the interaction was the key prediction, follow-up pairwise comparisons were also performed. While rehearsing failure stimuli, movements were faster in a backward than forward direction, *F* (1, 127) = 5.54, *p* = .02, PES = .04. On trials involving success words, by contrast, movements were directionally faster in a forward than backward direction, *F* (1, 127) = 2.46, *p* = .12, PES = .01.

There was no speed-accuracy tradeoff. Rather, a parallel Prime Category by Movement Direction interaction was observed for accuracy rates across the four conditions, F (1, 127) = 4.21, *p* = .04, PES = .03. Backward movements were somewhat more accurate following failure (M = 96.59%) than success (M = 96.03%) primes, whereas forward movements were somewhat more accurate following success (*M* = 88.74%) than failure (*M* = 87.03%) primes.

As with Experiment 1, the Prime Category by Movement Direction interaction remained significant when deleting trials involving the words advance, progress, backslide, and recede, which possess polysemy, *F* (1, 127) = 7.52, *p* < .01, PES = .06.

## Discussion

Experiment 2 removed some compatibility features likely present in Experiment 1. Movement responses were not contingent on word category and therefore relevant stimulus-response compatibility mechanisms [32] were absent. Also, participants did not perform a binary classification task on the primes, a feature that can result in stimulus-response structural overlap according to [40]. Indeed, participants in Experiment 2 merely rehearsed and repeated success versus failure words, a procedure akin to semantic priming [41]. Under such circumstances, it would not be surprising if the interaction of interest was reduced in magnitude, and it was. What had been a 57 ms cross-over interaction with a partial eta squared value of .21 became a 16 ms cross-over interaction with a PES value of .10. In addition, a combined data set revealed that there was a significant impact of Experiment on the Prime Category by Movement Direction interaction, as revealed by a three-way interaction among these factors, *F* (1, 202) = 9.39, *p* < .01, PES = .04. Nonetheless, success and failure words did prime forward versus backward movements in Experiment 2, results furthering the idea that these concepts are grounded in simulations of directional movement.

## General Discussion

In the seemingly pervasive journey metaphor, our life’s goals are ahead of us and moving backward represents going in the wrong direction [21]. Similar dynamics are thought to underlie our conceptions of self-regulation [26]. These considerations suggest that people may ground their ideas about success and failure, at least in part, in simulations of directional movement. If so, such motoric simulations should be apparent in priming paradigms [6]. This novel hypothesis was tested in two experiments. Primes consisted of success or failure words, but the dependent measure consisted of actual movements in a forward or backward direction. Both experiments found a cross-over interaction such that success words sped forward (versus backward) movements, whereas failure words sped backward (versus forward) movements. Together, the two experiments converge on a novel phenomenon of significance to the self-regulation and embodied cognition literatures.

Although the forward-backward dimension of movement has received some attention, it has arguably received less attention than deserved. These movements are so basic in nature that they appear to have self-regulatory significance in army ants, flatworms, and even paramecia [42]. Whether such movements “scale up” to the more abstract ways that human beings manage themselves has been less certain, but advocates for this position can be found [25,43]. Viewed from this perspective, the present findings are important because they attribute meaning to these movements, meaning that accords with more impressionistic perspectives of nonverbal behavior [24].

Even so, the primary purpose of the investigation was to extend our knowledge of embodied cognition. There are many ways to succeed or fail and success and failure must necessarily be somewhat abstract concepts for this reason [20]. Our priming manipulations reinforced the abstract nature of the concepts in that there were no personal experiences of success or failure involved and primes consisted of individual words. Despite these rather minimal conditions, we followed conceptual metaphor theory [14] in thinking that people ground these concepts in terms of more concrete sensory-motor experiences, particularly those related to forward versus backward motion [23]. That the findings were in accord with hypotheses extends the embodied cognition perspective to concepts and processes that had hitherto not been documented.

Experiment 1 had some procedural features that took advantage of possible stimulus-response compatibility mechanisms [32]. This was intentional because such features (such as making movements to categorize stimuli) are thought to result in more sensitive and valid paradigms [31,44]. Nonetheless, it also seemed useful to examine the scope of our phenomenon and Experiment 2 did so by removing features that are believed to contribute to stimulus-response compatibility [39–40]. For example, movement direction was not contingent on the nature of the primes in the second experiment. That there was a reliable interaction under these circumstances, we think, argues in favor of a representational locus for at least some of the processes examined. This account could be strengthened, however, perhaps by the use of the ERP technology [12]. We might expect success primes, for example, to facilitate perceptions of visually depicted forward movements, as revealed by early components of the ERP waveform (following Teuscher, McGuire, Collins, & Coulson [45]).

Our priming procedures were consistent with the embodied cognition literature [11,30], but one stimulus issue should be discussed. Metaphors often work through polysemy [15] and there was polysemy to a small minority of our stimulus words. Specifically, of the 20 word stimuli, two (“advance” & “progress”) could refer to actual forward motion and two (“backslide” & “recede”) could refer to actual backward motion. Our results were not, however, dependent on the use of these words in that prime type by movement direction interactions remained robust when deleting trials involving these word stimuli, *p*s < .01 (see footnotes 3 & 5). Possible carryover effects also seem unlikely. These prime stimuli would not differentially alert participants that forward and backward movements were of interest because the tasks actually required forward or backward movements quite irrespective which word primes were presented. These prime stimuli could not help participants anticipate movement directions for other trials because words and/or movement directions were randomly assigned at the trial level. And these prime stimuli could not favor forward or backward interpretations for the remaining stimuli because the remaining stimuli did not possess polysemy of this type. Altogether, then, the findings cannot be attributed to the use of a small minority of primes that possessed some degree of polysemy.

As another discussion point, it is useful to note that forward and backward movements are also used in approach-avoidance studies [46]. In these studies, the idea is that people may be automatically predisposed to seek appetitive objects (e.g., friends) while seeking to avoid aversive ones (e.g., enemies). The present phenomenon is different. One does not interact with success and failure in the same way that one interacts with appetitive or aversive objects; rather, success and failure are outcomes rather than objects. Because this is the case, success and failure necessarily have an abstract quality to them [46] and the relevant processes are distinct [26]. Consider contextual factors in this connection. Whether forward movements signify approach or avoidance can be altered somewhat dramatically by prefatory instructions or by the spatial layout of the task [47–48]. A similar modulation by contextual factors is less likely to characterize associations linking concepts of success and failure to simulations of forward versus backward movement [14,21–22].

Other interesting studies have linked forward and backward movements to conceptions of the past and future. For example, Miles, Nind, and Macrae [49] asked people to recall their lives four years in the past or to imagine their life four years in the future. When thinking about the future (past), participants increasingly swayed in a forward (backward) direction over the course of 15 seconds. This is a very interesting result, but success and failure are momentary occurrences that cannot be equated with the past and the future. In this connection, we suggest that the forward/backward dimension is recruited for multiple distinct purposes including representations of time [49–50] and of success and failure (current results). That the forward/backward dimension is used for multiple representation purposes makes sense in that this dimension is such a fundamental one to the body and its interactions with the environment [14,50].

### Caveats and Conclusions

We thought it useful to present visual feedback to guide joystick movements [51]. Somewhat naturally, forward movements displaced the joystick cursor upward and backward movements displaced it downward [33]. As noted by an astute reviewer, this procedural detail may have introduced verticality associations that could be controlled in future studies. Future studies might also examine whether forward or backward movements prime success or failure concepts. Directionality information of this type is useful in better understanding the nature of embodied associations (e.g., [52]). In fact, we hope that the current findings inspire a number of directions for future research. Regardless, though, the results suggest that the concepts of success and failure are grounded in perceptual-motor simulations [11] of forward versus backward movement.
